# Carotid Pseudoaneurysm Repair in a Patient with Myeloproliferative Disorder Complicated by Contralateral Stroke, Graft Thrombosis, Pyoderma Gangrenosum, and Superinfection: A Case Report

**DOI:** 10.3390/jcm15072768

**Published:** 2026-04-06

**Authors:** Cristian Voica, Dan Alexandru Cercel, Maria Sabina Safta, Bogdan Popescu, Iulian Tiboaca, Cristina Dumitru, Gabriel-Petre Gorecki, Bogdan Severus Gaspar, Anca Mihaela Cîrtog, Horatiu Moldovan

**Affiliations:** 1 Faculty of Medicine, Carol Davila University of Medicine and Pharmacy, 050474 Bucharest, Romania; cristian.voica@drd.umfcd.ro (C.V.); mariasabinasafta@gmail.com (M.S.S.); bogdan-stefan.popescu@outlook.com (B.P.); tiboaca.iulian@gmail.com (I.T.); dumitru.mihaela.cristina@gmail.com (C.D.); bogdan.gaspar@umfcd.ro (B.S.G.); h_moldovan@hotmail.com (H.M.); 2 Department of Cardiovascular Surgery, Emergency Clinical Hospital Bucharest, 014461 Bucharest, Romania; 3 Department of Anesthesia and Intensive Care, Faculty of Medicine, Titu Maiorescu University, 040051 Bucharest, Romania; gabriel.gorecki@prof.utm.ro; 4 Department of General Surgery, Bucharest Clinical Emergency Hospital, 014461 Bucharest, Romania; 5 Department of Dermatology, Bucharest Clinical Emergency Hospital, 014461 Bucharest, Romania; acirtog@yahoo.com; 6 Academy of Romanian Scientists, 54, Spl. Independentei, 050711 Bucharest, Romania

**Keywords:** carotid pseudoaneurysm, pyoderma gangrenosum, myeloproliferative disorder, thrombocytosis, stroke, graft thrombosis, wound infection

## Abstract

Carotid pseudoaneurysms are rare and potentially life-threatening, often necessitating urgent surgical intervention. Patients with myeloproliferative disorders (MPD) are predisposed to thrombotic and inflammatory complications. Pyoderma gangrenosum (PG), a rare neutrophilic dermatosis, is often misdiagnosed in postoperative settings. In the following article, we present a case of a 58-year-old woman with Philadelphia-negative MPD, neutrophilic leukocytosis, thrombocytosis, osteoporosis, and hypothyroidism, who presented with a giant left common carotid artery pseudoaneurysm. She underwent urgent surgical revascularization via bypass using an autologous reversed saphenous vein graft from the right thigh and external carotid artery ligation. Immediately postoperatively, the patient developed left hemiparesis. Initial CT scans showed bypass graft occlusion and right MCA stroke. Immediate thrombolysis resulted in complete motor recovery, although the bypass remained occluded. On postoperative day 10, necrotic wound lesions developed, initially treated as infectious. After worsening post-debridement, dermatologic evaluation raised suspicion for PG, confirmed by biopsy. She responded well to corticosteroid therapy. Four weeks later, the thigh wound became superinfected with Pseudomonas aeruginosa and Klebsiella pneumoniae, successfully treated with broad-spectrum antibiotics. The patient fully recovered within two months. This case illustrates the complex interplay between vascular, thrombotic, and inflammatory complications in patients with MPD and emphasizes the importance of multidisciplinary care and early recognition of PG.

## 1. Introduction

Carotid artery pseudoaneurysms are uncommon but urgent vascular conditions, often resulting from trauma, infection, or surgical complications. Their management can be challenging, especially in patients with underlying hematologic disorders [1]. Myeloproliferative disorders (MPD) such as essential thrombocythemia or polycythemia vera predispose patients to thrombotic events due to elevated platelet counts, leukocytosis, and dysfunctional hematopoiesis [2]. These patients are at increased risk of perioperative complications, especially following vascular surgery [2].

Zhang et al. reported that between 40% and 50% of patients with MPD experience arterial or venous thrombotic events at some point during the course of their disease, with cardiovascular complications representing the primary cause of morbidity and mortality in this population [3]. Arterial thrombosis in patients with MPN carries serious clinical implications, being correlated with elevated mortality rates, a higher probability of disease progression to myelofibrosis, and an increased incidence of secondary cancers [4,5,6].

The presence of a myeloproliferative neoplasm as a comorbid condition elevates the risk of thromboembolic complications during cardiac surgery [7]. Elevated platelet counts in patients with myeloproliferative disorders confer a higher risk of perioperative hemostatic complications—such as bleeding, thrombosis, and thromboembolic events—underscoring the need for individualized perioperative assessment and management [8].

Pyoderma gangrenosum (PG) is a rare, noninfectious neutrophilic dermatosis that manifests as rapidly progressing superficial tissue damage. Its ulcerative type presents as rapidly developing, painful ulcers with irregular shapes, necrotic tissue, and undermined edges [9]. It is commonly associated with systemic diseases such as inflammatory bowel disease, rheumatoid arthritis, and hematologic disorders, including MPD [10,11]. In 25–50% of patients, a triggering event—such as surgery or physical trauma—can be identified [9]. Diagnosis is clinical and histological, with biopsy typically revealing sterile neutrophilic infiltration [10,11]. Surgical debridement should be avoided, as it often worsens PG due to pathergy. Despite its rarity, postoperative PG has been increasingly recognized in surgical patients, with some cases reporting superimposed infections as due to use of immunosuppressant therapy [12,13].

## 2. Case Report Description

**Figure 1:**
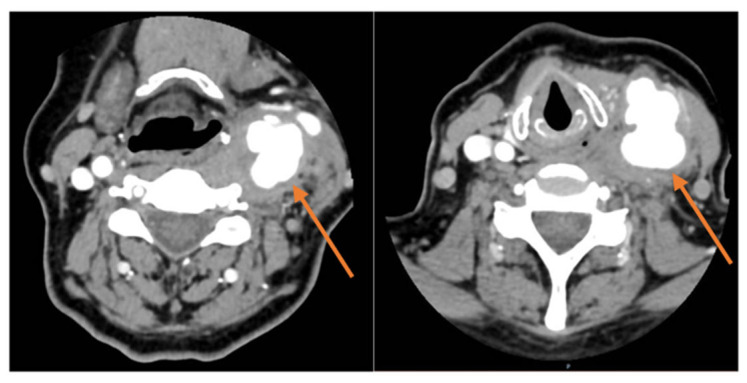
Giant left common carotid artery pseudoaneurysm on CT.

We present the case of a 58-year-old female with a known Philadelphia-negative MPD under treatment with hydroxycarbamide (500 mg/day), hypothyroidism (under Euthyrox treatment), and osteoporosis (under bisphosphonates) who came into our clinic with a tender, pulsatile left-sided neck mass and no neurological deficits. Laboratory workup showed thrombocytosis (875.50 × 10^3^/μL) and leukocytosis (34.28 × 10^3^/μL) with neutrophilia. From the patient history, we knew JAK2, CALR, and MPL mutation testing were negative, and the direct Coombs test was negative. The patient was admitted, and a CT scan was performed, resulting in a diagnosis of a giant left common carotid artery pseudoaneurysm, as shown in Figure 1.

**Figure 2:**
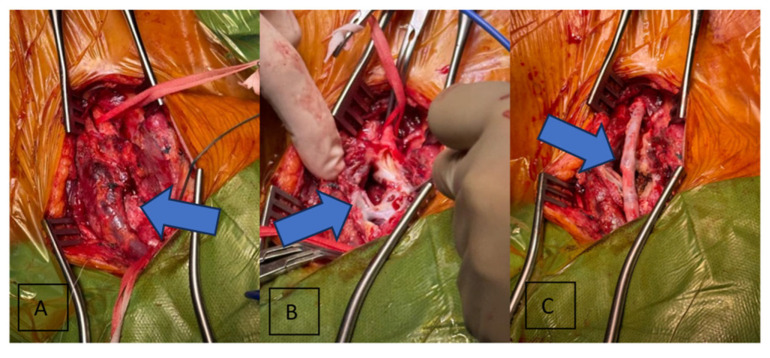
Intraoperative view: (**A**) the left common carotid artery after aneurysm isolation; (**B**) the exposure of the carotid pseudoaneurysm during surgical dissection; and (**C**) the completion of carotid bypass using a reversed saphenous vein graft from the right thigh.

The following day, the patient was taken to the operating room, where she was put under general anesthesia. Single-lumen intubation was used to secure the airways, as independent ventilation for each lung was not required for the proposed procedure. Standard incision at the cervical level was performed, and delicate dissection of the underlying tissue was done. The vagus and hypoglossal cranial nerves were identified and preserved. After dissecting nearby structures and having clear control of the upper and lower margins of the aneurysm, the common carotid and the internal carotid were clamped. After clamping the vessels, a large vertical incision into the mass was made, which exposed a rupture in the posterior side of the aneurysm. Because of the intensive destruction of tissue, it was considered that the best course of action would be to ligate the external carotid and perform a common-to-internal carotid bypass using an autologous saphenous vein graft, which had been harvested from the right thigh of the patient while the dissection of the tissue surrounding the carotid aneurysm was taking place. End-to-end anastomosis was used for the bypass. The vessels were declamped before completing the suture to ensure adequate blood flow and backflow were present, and to ensure no air embolism. After completing the suture, the bypass was functional with strong arterial flow present. Both surgical wounds were closed using intradermal suturing. During the surgery, the patient exhibited a stable heart rate with no cardiac rhythm abnormalities and was normotensive through the entire intervention. Figure 2 shows the intraoperative steps performed.

**Figure 3:**
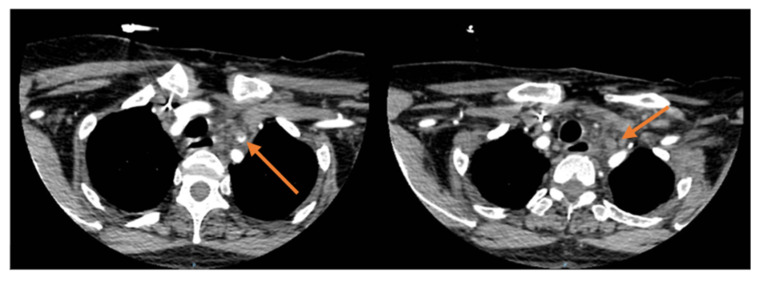
Postoperative CT scan showing thrombosis of the carotid bypass graft.

The patient was extubated immediately after being transported to the ICU, with no signs of motor or sensory impairment. The patient received 1 unit of blood, 75 mg of aspirin was given, and heparin treatment was initiated, but within the next few hours, the patient developed left-sided hemiparesis, and a CT scan performed at that time showed an acute right MCA stroke and carotid bypass occlusion, as seen in Figure 3. Immediate systemic thrombolysis was initiated, which led to full motor recovery, but the bypass graft could not be saved and remained occluded. This immediate postoperative graft thrombosis was attributed to the MPD-related hypercoagulable state, as during the surgical procedure, we ensured adequate flow through the graft was present. The histopathology test of the carotid tissue showed a medial arterial wall hypertrophy and diffuse sclerohyalinization.

**Figure 4:**
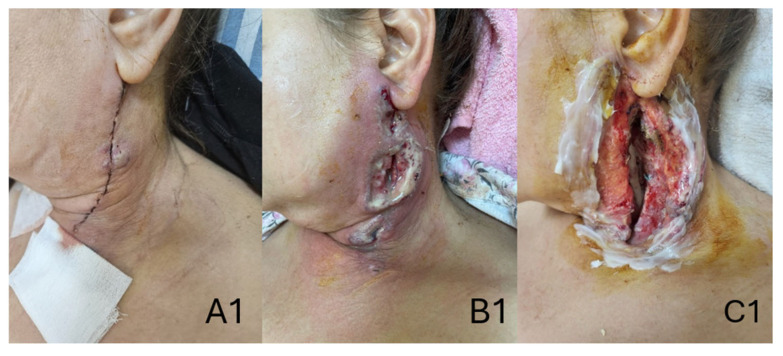

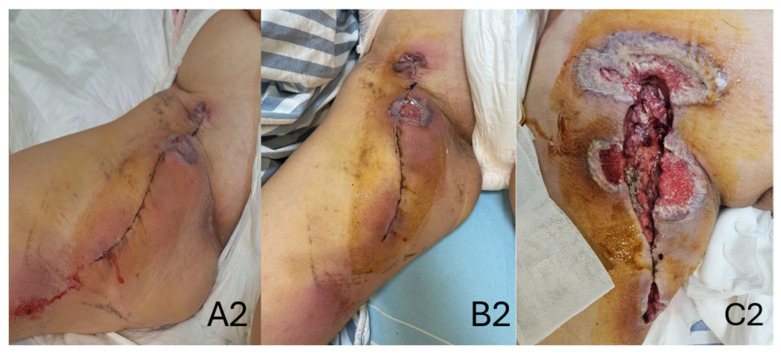
Local deterioration of the surgical sites. (**A1**,**A2**) on the 8th postoperative day; (**B1**,**B2**) the 11th postoperative day; and (**C1**,**C2**) post-surgical debridement wound on the 16th postoperative day.

The patient’s recovery progressed slowly over time, and we decided to keep the patient under supervision for a longer period of time due to the bypass graft postoperative complication. The next days followed a usual recovery process, as the surgical site incisions were recovering normally, but on the tenth postoperative day, a small bullous lesion with serous secretions in small quantities developed at the neck surgical site, and necrotic wound lesions developed on the right thigh. Immediate antibiotic treatment was initiated, and bacteriological probes were taken, but the result could not identify a specific infectious agent, classifying the probe as polymicrobial colonization. Under the antibiotic treatment, the lesions showed signs of worsening; thus, surgical debridement was performed after 3 days of poor local evolution. Following the surgical debridement, the lesions deteriorated even further. The local deterioration of the surgical sites is observable in Figure 4.

**Figure 5:**
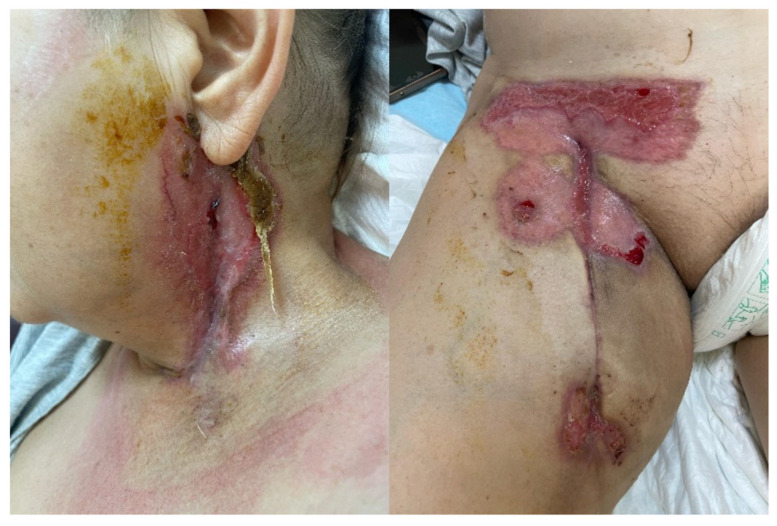
Local wound improvement during the 5th postoperative week.

The requested dermatology consult resulted in a cutaneous biopsy and initiation of empirical topical corticosteroid therapy. Biopsy results showed tissue fragment with acanthosis, papillomatosis, focal epidermic atrophy, and dermic edema with abundant mixed inflammatory infiltrate, predominantly granulocytes, confirming neutrophilic dermatosis, consistent with PG, leading to systemic corticosteroid treatment with 32 mg of prednisone daily, which led to significant improvement of the wounds, as seen in Figure 5. Furthermore, the local wound care consisted of wound cleaning every other day for weeks using saline solution to wash the wound, iodine to clean a large surface of the surrounding healthy skin, topical corticosteroid cream on the rough edges to prevent further spreading of the necrosis, silver dressings for bacterial infection prevention, and Grassolind mesh. These last two layers were also used to prevent the sterile gauze that was used to cover the wounds from sticking together, further damaging them.

**Figure 6:**
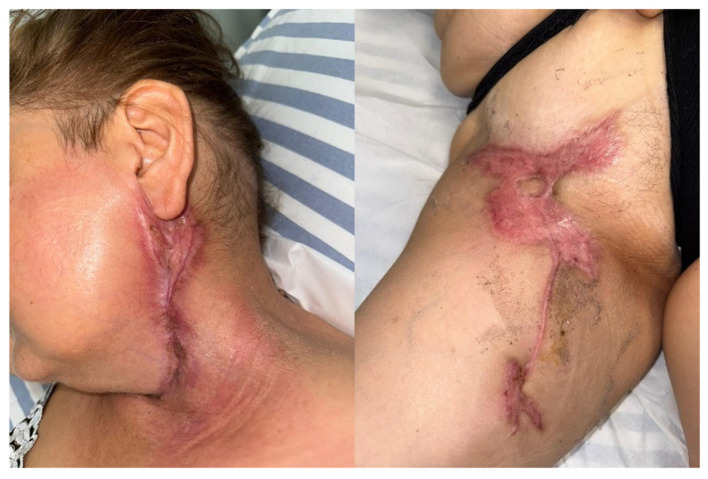
Healed surgical wounds on the 8th postoperative week.

By the fourth postoperative week, the surgical sites were almost healed with new epidermis forming on the wounds. On the fifth postoperative week, the thigh wound showed signs of superinfection, including green discharge and a foul odor. Cultures identified two different microbes present: Pseudomonas aeruginosa and Klebsiella pneumoniae, both antibiotic-sensitive. The patient received a 3-week course of triple antibiotics treatment with meropenem, tigecycline, and colistin. By week eight, all wounds had healed, as seen in Figure 6. No new pyoderma gangrenosum lesions were identified, and despite the persistent carotid bypass graft occlusion, the neurological function remained unaffected, probably due to collateral circulation.

After a long period of complications, the patient was successfully discharged and remained within our attention for follow-up. She regularly visited our outpatient ward, and at the 6-month follow-up, she had no neurological deficit with no new PG events.

## 3. Comprehensive Literature Review

Extracranial carotid artery aneurysms, be they true aneurysms or pseudoaneurysms, represent a rare but critical vascular pathology accounting for less than 1% of all arterial aneurysms, with the most usual presentation site being the common carotid bifurcation, followed by the middle to distal part of the internal carotid [14]. While aneurysms maintain the full structural integrity of the affected vessel, pseudoaneurysms are defined by a breach of the arterial wall, be it at the intimal level or throughout the entire structure, containing a pulsatile hematoma bounded by surrounding perivascular tissue. This structural distinction from true aneurysms underscores their typically acquired etiology, most commonly stemming from trauma, iatrogenic injury during surgical or endovascular procedures, or infection [14]. Accurate diagnosis hinges on a multi-modal imaging approach. Duplex ultrasound serves as an effective initial screening tool, often identifying the pathognomonic “to-and-fro” Doppler waveform. Computed tomographic angiography is the contemporary gold standard, providing more accurate details on aneurysm size, morphology, and relation to critical anatomical structures, which is essential for pre-procedural planning [15].

Management strategies for aneurysms are tailored to the patient’s anatomy and comorbidities, but mainly fall into three categories: open surgical repair, endovascular therapy, or medical therapy. Open techniques, such as resection with interposition grafting using autologous vein or prosthetic material, are still considered the definitive treatment, especially when dealing with true aneurysms, considering their association with conjunctive tissue disease [14]. Endovascular options, primarily stent–graft placement or coil embolization, offer a minimally invasive alternative for high-surgical-risk patients and are the preferred method in small, asymptomatic pseudoaneurysms [14,16,17]. The choice between these definitive treatment options requires careful consideration of the aneurysm’s characteristics and the patient’s overall health.

Both short-term and long-term complications, although rare, significantly impact outcomes. In the immediate perioperative period, the most important complications are thromboembolic or hemorrhagic stroke and early graft thrombosis. Injury of the cranial nerves in carotid surgery remains a possibility, most often affecting the vagus nerve, followed by the hypoglossal nerve, often without long-term complications and full recovery in over 99% of patients in 6–12 months [18]. Long-term risks include graft infection, stenosis, late thrombosis, and the rare recurrence of the pseudoaneurysm. In the Leicester series, freedom from severe stenosis or graft occlusion is seen in over 92% of patients at 6 months, 86% of patients at 12 months, and 83% at 24 months, for which, excluding one asymptomatic patient, carotid angioplasty was performed [19]. Considering the thromboembolic event risk and the stenosis risk, follow-up should be considered for all patients.

### 3.1. Myeloproliferative Disorders and Association with Vascular Surgical Procedures

Myeloproliferative disorders create a profound hypercoagulable state that poses a significant challenge in all vascular surgeries. The pathophysiological triad of elevated blood cell counts, aberrant platelet activation, and dysfunctional hematopoiesis predisposes patients to both arterial and venous thrombotic events, which are a leading cause of their morbidity and mortality despite adequate treatment [4]. Studies have shown the association between arterial thrombotic events and progression of polycythemia vera and essential thrombocythemia towards myelofibrosis or development of secondary tumors [4]. In the context of vascular surgery, this pro-thrombotic state drastically elevates the perioperative risk of graft thrombosis, intimal hyperplasia, and subsequent occlusion, complicating what might otherwise be a technically successful procedure.

The development of early or late carotid graft thrombosis is a potentially fatal complication, and patients with underlying MPDs are disproportionately vulnerable. The pro-thrombotic state can overwhelm standard anticoagulation protocols, leading to acute graft failure and ischemic stroke [20]. Consequently, the management of these patients demands a proactive, multidisciplinary strategy involving hematologists for pre-operative cytoreduction (e.g., with hydroxyurea), meticulous perioperative anticoagulation bridging, and lifelong antiplatelet therapy to mitigate this inherent risk and maximize graft patency [21,22].

### 3.2. Pyoderma Gangrenosum and Association with Surgical Procedures

Pyoderma gangrenosum is a rare, neutrophilic dermatosis characterized by painful, rapidly progressing ulcers with undermined violaceous borders. Although it is often associated with systemic inflammatory conditions, including inflammatory bowel disease, rheumatoid arthritis, and, most frequently, hematologic disorders such as MPDs, it has been shown to appear idiopathically with surgical procedures as a trigger [12,23].

The traits of PG make it a particularly devastating complication in the postoperative setting. A wound that fails to heal or rapidly deteriorates after surgery is often misdiagnosed as a necrotizing soft tissue infection. This misdiagnosis leads to the standard of care for infection: aggressive surgical debridement. However, in PG, debridement acts as further trauma, invoking pathergy and starting a dramatic expansion of the ulceration, resulting in significant tissue loss [23]. In a review of clinical cases, Tolkachjov SN et al. have shown that in 90% of cases, the first line of treatment used is antibiotics, and over 70% of patients underwent surgical debridement [12]. A high index of suspicion is required in at-risk patients, and diagnosis often requires histopathological examination to exclude infection. First-line treatment consists of systemic immunosuppression to control the underlying neutrophilic inflammation. Any suspicion of PG in a postoperative wound warrants immediate dermatology consultation to prevent iatrogenic harm, misdiagnosis and guide appropriate immunosuppressive therapy in case of diagnosis confirmation [24,25].

Differential diagnosis in pyoderma gangrenosum is often difficult. Differentiation is necessary from ulcers due to circulatory disorders; ulcers due to thrombosis; ulcers due to malignant neoplasms, such as squamous cell carcinoma, basal cell carcinoma, and malignant lymphoma; ulcers due to vasculitis, such as polyarteritis nodosa, rheumatoid vasculitis, and granulomatous vasculitis; and ulcers due to infections, such as deep mycosis, non-tuberculous mycobacteriosis, and necrotizing fasciitis. While biopsy is crucial for diagnosis, excluding vasculitis histologically can be particularly challenging. It may show features that mimic vasculitis, such as neutrophil and mononuclear cell infiltration around dermal blood vessels, along with fibrin deposition in the vascular walls; however, these are often secondary to the underlying skin ulcer (a phenomenon known as pseudo-vasculitis) rather than true vasculitis. Even with a skin biopsy, achieving a definitive diagnosis can remain difficult, as the results can vary depending on the biopsy site and timing, potentially requiring samples from multiple locations [26].

Treatment in cases of ulcerative lesions on the lower leg, including pyoderma gangrenosum (PG), centers on rest. These lesions are unlikely to heal effectively if weight-bearing on the lower legs is not reduced. Consequently, PG management often requires hospitalization to facilitate proper rest. Hospitalization is recommended for comprehensive evaluation, treatment, and pain control in PG and other similar conditions, regardless of the ulcers’ location, size, or number. For topical therapy, standard ulcer care applies: maintaining a moist environment, applying appropriate topical agents and dressing materials, protecting the skin, and using bandages to alleviate edema [26].

Oral treatments for pyoderma gangrenosum (PG) are highly recommended, with corticosteroids such as prednisolone receiving a grade A recommendation for their effectiveness in managing ulcerative lesions, particularly when topical therapies alone are insufficient. Treatment typically begins with prednisolone at 20 mg/day or 0.5 mg/kg, escalating to 1 mg/kg for severe cases, and may be combined with cyclosporine (3–5 mg/kg) for unresponsive or relapsing conditions, though cyclosporine is used off-label in some contexts. The STOP GAP trial demonstrated no significant differences between prednisolone (up to 75 mg/day) and cyclosporine (up to 400 mg/day) in ulcer area reduction after 6 weeks, cure rates after 6 months, or recurrence rates, indicating comparable efficacy. For immunosuppressants, cyclosporine also holds a grade A recommendation due to its rapid effects and strong evidence from cohort studies and systematic reviews, making it a viable first- or second-line option alongside corticosteroids, with other immunosuppressants rated C1-C2. Overall, drug selection should be tailored to individual cases, considering profiles like cost-effectiveness, as both approaches require extended treatment durations (up to 6 months for cure) and highlight the need for more advanced therapies [26].

## 4. Discussion

This case highlights the multifactorial complexity of treating patients with MPD who require vascular surgery. The increased thrombotic risk posed by MPD was evident in the early graft thrombosis, despite appropriate surgical technique. Arai et al. reported a case of a patient with JAK2-positive MPD (essential thrombocythemia) who developed in-stent thrombosis and subsequent stroke following carotid artery stenting, underscoring the significant thrombotic risk associated with myeloproliferative disorders during vascular interventions. Their findings emphasize that even with technically successful procedures and standard antithrombotic therapy, patients with MPD remain vulnerable to early thrombotic occlusion due to persistent hematologic hypercoagulability [27]. Immediate thrombolysis proved effective in reversing neurological symptoms, a fortunate outcome that is not always guaranteed.

PG is a diagnostic challenge, especially in the postoperative setting, where wound complications are often presumed to be infectious. In this case, empirical antibiotic therapy and debridement initially delayed appropriate treatment. The subsequent biopsy and dermatologic assessment were crucial in identifying PG. Systemic and local corticosteroid therapy was initiated promptly after the diagnosis, leading to rapid improvement of both the neck and thigh lesions. This underscores the importance of early recognition and initiation of immunosuppressive therapy in postoperative PG, as delays can result in extensive tissue damage and prolonged recovery.

The subsequent superinfection with Pseudomonas aeruginosa and Klebsiella pneumoniae required broad-spectrum antibiotics, illustrating the vulnerability of immunosuppressed patients with extensive postoperative wounds. The occurrence of superinfection in PG wounds, although infrequent, is clinically significant and can lead to delayed healing and systemic infection. Aggressive antibiotic therapy tailored to culture sensitivities was essential for wound resolution in this patient. This case emphasizes that postoperative PG may mimic or coexist with infectious complications, complicating clinical decision-making.

Surgical interventions, such as debridement, may exacerbate PG due to pathergy, making conservative wound care with topical corticosteroids and gentle dressings essential alongside systemic therapy. Furthermore, in patients with underlying hematologic disorders like MPD, additional factors—including thrombocytosis, leukocytosis, and hypercoagulability—may contribute both to thrombotic complications and impaired wound healing, as demonstrated by the carotid bypass graft thrombosis in this patient.

Moreover, this article emphasizes the need for both physicians and patients to be well-informed, even in what seems a routine case, about all possible complications. Patients’ pathologic history remains a crucial initial step in the anamnesis of patients, as it brings information that helps the doctor to make a correct differential diagnosis.

Furthermore, this case highlights once again the need for a multidisciplinary approach in the management of our patients; in this case, the collaboration of cardiovascular surgery, intensive care, anesthesiology, dermatology, and pathology specialists managed to cure this patient.

## 5. Conclusions

We present a complex clinical case where an urgent vascular pathology presents multiple infectious and inflammatory complications that were successfully treated using a multidisciplinary approach. The coexistence of a myeloproliferative disorder, with its associated hypercoagulable state, further increased the risk of vascular complications and graft thrombosis.

This case underscores the importance of considering PG in postoperative patients who develop atypical wound ulcerations, particularly in the setting of underlying hematologic disorders, and emphasizes that early recognition, prompt dermatologic evaluation, and coordinated multidisciplinary management are critical to prevent disease progression, minimize tissue damage, and optimize overall patient outcomes.

Early recognition of PG and timely initiation of immunosuppressive therapy, in conjunction with close collaboration between vascular surgeons, dermatologists, and infectious disease specialists, are critical for optimizing outcomes. A multidisciplinary approach not only facilitates accurate diagnosis and targeted treatment but also reduces the risk of unnecessary surgical interventions that may exacerbate PG through pathergy. Ultimately, proactive management of the underlying hematologic disorder, combined with coordinated postoperative care, is essential to prevent complications, promote wound healing, and improve long-term prognosis in this challenging patient population.

## Data Availability

Data sharing is not applicable to this article as no new data were created or analyzed in this study.

## Abbreviations

The following abbreviations are used in this manuscript:

MPD	Myeloproliferative Disorders
PG	Pyoderma Gangrenosum
